# Urinary extracellular vesicle RNAs as novel **biomarkers** for diagnosis and prognosis of lupus nephritis

**DOI:** 10.1093/ckj/sfaf295

**Published:** 2025-09-22

**Authors:** Heng Zhang, Xuexue Zheng, Saisai Huang, Li Qian, Junxiang Wu, Zhongtian Shi, Meng Jia, Yang Bai, Longwei Jiang, Shaochang Jia, Ke Zen, Yanggang Yuan, Jun Liang, Hongwei Liang

**Affiliations:** Department of Emergency, Nanjing Drum Tower Hospital, School of Life Science and Technology, China Pharmaceutical University, No. 24, Tongjiaxiang, Gulou District, Nanjing, China; Department of Emergency, Nanjing Drum Tower Hospital, School of Life Science and Technology, China Pharmaceutical University, No. 24, Tongjiaxiang, Gulou District, Nanjing, China; Department of Rheumatology and Immunology, Nanjing Drum Tower Hospital, Medical School of Nanjing University, No. 22 Hankou Road, Gulou District, Nanjing, China; Department of Nephrology, The First Affiliated Hospital of Nanjing Medical University, No.140 Hanzhong Road, Gulou District, Nanjing, China; Department of Emergency, Nanjing Drum Tower Hospital, School of Life Science and Technology, China Pharmaceutical University, No. 24, Tongjiaxiang, Gulou District, Nanjing, China; Department of Emergency, Nanjing Drum Tower Hospital, School of Life Science and Technology, China Pharmaceutical University, No. 24, Tongjiaxiang, Gulou District, Nanjing, China; Zhenjiang Kang Cetong Technology Co., Ltd, Jurong, China; Zhenjiang Kang Cetong Technology Co., Ltd, Jurong, China; Institute of Geriatric Medicine, Jiangsu Province Geriatric Hospital, No. 65 Jiangsu Road, Gulou District, Nanjing, Jiangsu, China; Institute of Geriatric Medicine, Jiangsu Province Geriatric Hospital, No. 65 Jiangsu Road, Gulou District, Nanjing, Jiangsu, China; Department of Emergency, Nanjing Drum Tower Hospital, School of Life Science and Technology, China Pharmaceutical University, No. 24, Tongjiaxiang, Gulou District, Nanjing, China; Department of Nephrology, The First Affiliated Hospital of Nanjing Medical University, No.140 Hanzhong Road, Gulou District, Nanjing, China; Department of Rheumatology and Immunology, Nanjing Drum Tower Hospital, Medical School of Nanjing University, No. 22 Hankou Road, Gulou District, Nanjing, China; Department of Emergency, Nanjing Drum Tower Hospital, School of Life Science and Technology, China Pharmaceutical University, No. 24, Tongjiaxiang, Gulou District, Nanjing, China

**Keywords:** active lupus nephritis, machine learning analysis, RNA, RT‒qPCR, urinary extracellular vesicles

## Abstract

**Background:**

Lupus nephritis (LN) is among the most serious organ manifestations of systemic lupus erythematosus (SLE), contributing significantly to morbidity and long-term renal outcomes. The development of noninvasive biomarkers capable of distinguishing active LN from non-renal SLE is of considerable clinical importance. Although renal biopsy remains the diagnostic gold standard, its invasive nature limits its utility for serial monitoring. In recent years, urine has emerged as a promising noninvasive medium for detecting renal inflammation and assessing disease activity.

**Methods:**

This study investigated whether RNA signatures within urinary extracellular vesicles (EVs) could serve as diagnostic biomarkers for LN. Urinary EVs were isolated from 27 patients with active LN and 13 with LN in remission. RNA sequencing was conducted, and four candidate transcripts were prioritized using three independent machine learning algorithms. These candidates were subsequently validated in an independent cohort comprising 143 urine samples using TaqMan-based quantitative PCR with reverse transcription (RT–qPCR).

**Results:**

Among the identified candidates, LINC01127, RUNDC3A-AS1, and LRRN3 emerged as potential diagnostic biomarkers for LN. Notably, RUNDC3A-AS1 and LRRN3 demonstrated robust discriminatory capacity between proliferative (class III/IV) and non-proliferative (class V) forms of LN.

**Conclusions:**

Our findings identify urinary extracellular vesicle RNAs-particularly LINC01127, RUNDC3A-AS1, and LRRN3-as novel, noninvasive biomarkers with potential clinical utility for the diagnosis and subclassification of LN.

## GRAPHICAL ABSTRACT

**GRAPHICAL ABSTRACT:**
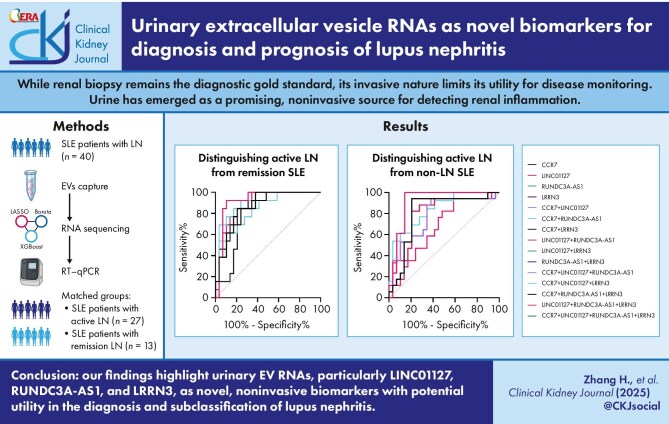

KEY LEARNING POINTS **What was known:**

- LN is a serious complication of systemic lupus erythematosus (SLE), often requiring kidney biopsy for diagnosis and classification.
- Urinary extracellular vesicles (uEVs) are a promising source of noninvasive biomarkers, with most studies to date focusing on small RNAs such as microRNAs.
- The clinical utility of long RNAs in urinary EVs as diagnostic markers for LN has been largely unexplored.

**This study adds:**

- Identification of three long RNAs—LINC01127, RUNDC3A-AS1, and LRRN3—in urinary EVs as candidate noninvasive biomarkers for active LN.
- Validation of these RNAs in an independent SLE cohort using RT–qPCR, demonstrating their diagnostic relevance.
- Evidence that RUNDC3A-AS1 and LRRN3 can discriminate between proliferative (class III/IV) and membranous (class V) LN, supporting their potential role in LN subclassification.

**Potential impact:**

- This study supports the development of urinary EV-derived long RNAs as a novel class of biomarkers for diagnosing and subclassifying LN.
- This study provides a noninvasive alternative to kidney biopsy for disease monitoring, with potential applications in clinical decision-making and personalized treatment strategies.

## INTRODUCTION

Lupus nephritis (LN) represents a severe clinical manifestation of systemic lupus erythematosus (SLE) and is a leading cause of disease-related morbidity, frequently culminating in progressive renal impairment. Approximately 60% of individuals with SLE develop LN, with 10%–15% advancing to end-stage renal disease (ESRD) [1]. Renal biopsy remains the diagnostic gold standard, enabling histopathological classification into ISN/RPS classes I–VI, which informs both therapeutic strategies and prognostic evaluation. Nevertheless, its invasive nature and susceptibility to sampling bias constrain its utility for routine or early disease monitoring.

Timely diagnosis and intervention are critical to altering the course of LN; however, existing diagnostic modalities lack sufficient sensitivity for early-stage detection [2]. This limitation has prompted growing interest in the identification of reliable, noninvasive biomarkers. Encapsulated by a lipid bilayer, extracellular vesicles (EVs) safeguard their nucleic acid and protein cargo from enzymatic degradation, thereby enabling stable intercellular communication through the transfer of RNAs, DNAs, and proteins. Emerging evidence suggests that exosomal RNAs may serve as promising diagnostic biomarkers across several diseases, including LN [3–5]. To date, most LN-focused studies of EVs have concentrated on small RNA species such as microRNAs and tRNA-derived small RNAs [3–5]. Notably, a urinary EV-based diagnostic assay targeting three long RNAs has been developed to distinguish benign from malignant prostate lesions [6], indicating that long RNA species may hold greatly diagnostic potential in LN. Nonetheless, the broader clinical application of RNA biomarkers in the EVs remains constrained by the lack of simple, efficient platforms for EV isolation and RNA profiling. Current methods are often labor-intensive, time-consuming, and not readily scalable.

In previous studies, we [7–9] and Li *et al.* [10] developed a novel method for EV isolation using wheat germ agglutinin (WGA)-conjugated magnetic beads, providing a more practical and scalable alternative to conventional techniques [7–10]. In the present study, we applied this WGA-based approach to the urinary EV compartment and coupled it with RT–qPCR for the quantification of long RNAs. Using this platform, we identified three long RNA species-LINC01127, RUNDC3A-AS1, and LRRN3-as candidate urinary EV-derived biomarkers for the diagnosis of LN. These findings support a noninvasive and potentially early detection strategy for LN, with implications for improving disease monitoring and clinical decision-making.

## MATERIALS AND METHODS

### Experimental design

To identify the biomarker in uEVs for LN diagnosis, we first performed a comprehensive RNA sequencing of uEVs from SLE (including 27 patients with active LN and 13 with LN in remission) isolated by WGA-coupled magnetic beads as previously studies [7–10]. Subsequently, three machine learning algorithms—least absolute shrinkage and selection operator (LASSO) [11], XGBoost [12], and Boruta [13]—were employed to assess the diagnostic potential of the dysregulated RNAs in the uEVs. Immediately, the selected RNAs in the uEVs were validated using RT–qPCR in 143 patients with SLE (89 patients with active LN, 41 with LN in remission, and 13 without renal involvement).

### Clinical samples

A total of 183 patients with SLE were recruited from Nanjing Drum Tower Hospital, Jiangsu Province Geriatric Hospital, and The First Affiliated Hospital of Nanjing Medical University. The cohort included 116 patients with active LN, 54 with LN in remission, and 13 without renal involvement. Diagnoses of SLE and LN were made in accordance with the Kidney Disease: Improving Global Outcomes (KDIGO) Clinical Practice Guideline for Glomerulonephritis [14]. Active LN was defined by impaired renal function accompanied by proteinuria (>0.5 g/day or >1.0 g/day if baseline proteinuria was present), active urinary sediment (hematuria and/or cellular casts), or biopsy-proven active glomerulonephritis. Remission of LN was defined by proteinuria <0.5 g/day, inactive urinary sediment, and stable renal function, indicated by normal serum creatinine and estimated glomerular filtration rate (eGFR). Renal biopsy specimens were evaluated using the 2003 International Society of Nephrology/Renal Pathology Society (ISN/RPS) classification, revised in 2018 [15], and the chronicity index (CI) was calculated as the sum of scores for glomerular sclerosis, fibrous crescents, tubular atrophy, and interstitial fibrosis. Urine samples from all patients were obtained on the day of renal biopsy or at presentation before immunosuppressive treatment in all patients in the active phase. Urine from 20 healthy control participants, matched for gender and age, were used as normal controls. All participants provided written informed consent, and the study protocol was approved by the Ethics Committees of all participating institutions. The demographics and clinical characteristics of the study participants are shown in Table S1.

### Isolation of uEVs by WGA-conjugated magnetic beads

For western blotting, nanoparticle tracking analysis (NTA), and transmission electron microscopy (TEM), 1 ml of urine was centrifuged at 3000×*g* for 10 minutes and filtered through 0.45-µm membranes (Millipore, HAWP04700) to remove the cell debris and large EVs (>0.5 nm). The filtrate was incubated with WGA-conjugated magnetic beads at room temperature for 1 hour. Bead-bound EVs were then separated using a magnetic rack, the supernatant was discarded, and the bead-bound EVs were washed twice with phosphate-buffered saline (PBS).

### Characterization of uEVs

The EVs were eluted using 500 mM *N*-acetyl-D-glucosamine (GlcNAc) in PBS from the bead-bound EVs. The procedure included: (i) washing 20 µl of beads three times with 400 µl of PBS; (ii) elution with 400 µl of GlcNAc buffer per 20 µl of beads, followed by 15-minute rotation at room temperature; (iii) magnetic separation of beads; and (iv) collection of the eluate for downstream analyses. Western blotting was performed using antibodies against CD63 (52090S, CST), TSG101 (28283–1-AP, Proteintech), ALIX (12422–1-AP, Proteintech), and CALNEXIN (10427–2-AP, Proteintech), EOGT (Proteintech, 27595‐1‐AP), NDST1 (Proteintech, 26203‐1‐AP), and PIGK (Abcam, ab201693) with HRP-conjugated secondary antibodies (ab205718, Abcam) for detection [7–9].

### RNA isolation

EVs were lysed with TRIzol reagent (Invitrogen, USA) and incubated at room temperature for 10 minutes. Subsequently, 200 µl of chloroform was added, followed by an additional 10-minute incubation. Samples were centrifuged at 12 000×*g* for 30 minutes at 4°C, and the resulting aqueous phase was transferred to a new tube and mixed with an equal volume of isopropanol. To enhance RNA precipitation, samples were incubated at−-20°C for 1 hour, then centrifuged at 12 000*×*g for 20 minutes at 4°C. The RNA pellets were washed with 75% ethanol, air-dried, and resuspended in 20 µl of nuclease-free water. RNA concentration and purity were assessed using a OneDrop-2000 spectrophotometer (NanoDrop Technologies).

### RNA sequencing

The total RNA of the EVs (isolated from 5 ml of urine by 50 µl of WGA-conjugated magnetic beads) was extracted using TRIzol reagent (Invitrogen). RNA quality and quantity were assessed using the NanoDrop 8000 UV-Vis spectrophotometer (NanoDrop Technologies, Wilmington, DE, USA) and the Agilent 4200 TapeStation (Agilent Technologies, Santa Clara, CA, USA). RNA libraries were prepared using the TruSeq^®^ RNA Access Library Prep Kit (Illumina, San Diego, CA, USA) following the manufacturer’s protocol. Sequencing was performed using the 100-bp paired-end mode on an Illumina HiSeq 2500 platform with the TruSeq Rapid PE Cluster and SBS kits. Sequencing reads were aligned to the human reference genome (hg19) using STAR, and gene expression was quantified via RNA-Seq by Expectation-Maximization. Quality control metrics were generated using RNA-SeQC. Gene fusion events were predicted using multiple algorithms, including ChimeraScan, deFuse, FusionMap, MapSplice, and TopHat. Expression levels were normalized using the transcripts per million method for cross-sample comparisons.

### Quantitative PCR with reverse transcription 

Total RNA was extracted from EVs isolated from 1 ml of urine using 10 μl of WGA-conjugated magnetic beads, employing TRIzol reagent (Invitrogen, USA) according to the manufacturer’s instructions. RNA concentration and purity were assessed using a OneDrop-2000 spectrophotometer (NanoDrop Technologies, USA). Complementary DNA (cDNA) was synthesized using the 1st Strand cDNA Synthesis Kit (Vazyme Biotech, China), and RT–qPCR was performed in 96-well plates using the ChamQ Universal SYBR qPCR Master Mix (Vazyme Biotech, China). U6 small nuclear RNA was used as an internal control. For cDNA synthesis, 8 μl of total RNA was mixed with 2 μl of 5 × gDNA Wiper Mix and incubated at 42°C for 2 minutes to remove genomic DNA. The reaction was then supplemented with 2 μl of 10 × RT Mix, 2 μl of HiScript III Enzyme Mix, 1 μl of random hexamers, and 5 μl of RNase-free water, followed by incubation at 37°C for 45 minutes. RT–qPCR was conducted using 10 μl of 2 × ChamQ SYBR qPCR Master Mix, 1 μl of gene-specific primer mix, and the synthesized cDNA, under the following cycling conditions: initial denaturation at 95°C for 30 seconds, followed by 40 cycles of 95°C for 10 seconds and 60 °C for 30 seconds. All primers were synthesized by Thermo Fisher Scientific (USA).

### Statistical analysis

Statistical analyses were performed using GraphPad Prism v.8.0 (GraphPad Software, USA). Comparisons between groups were conducted using Student’s *t*-test or one-way ANOVA followed by Tukey’s *post hoc* test. A *P* value <.05 was considered statistically significant.

## RESULTS

### Characterization of EVs Isolated via WGA-coupled magnetic beads

**Figure 1:**
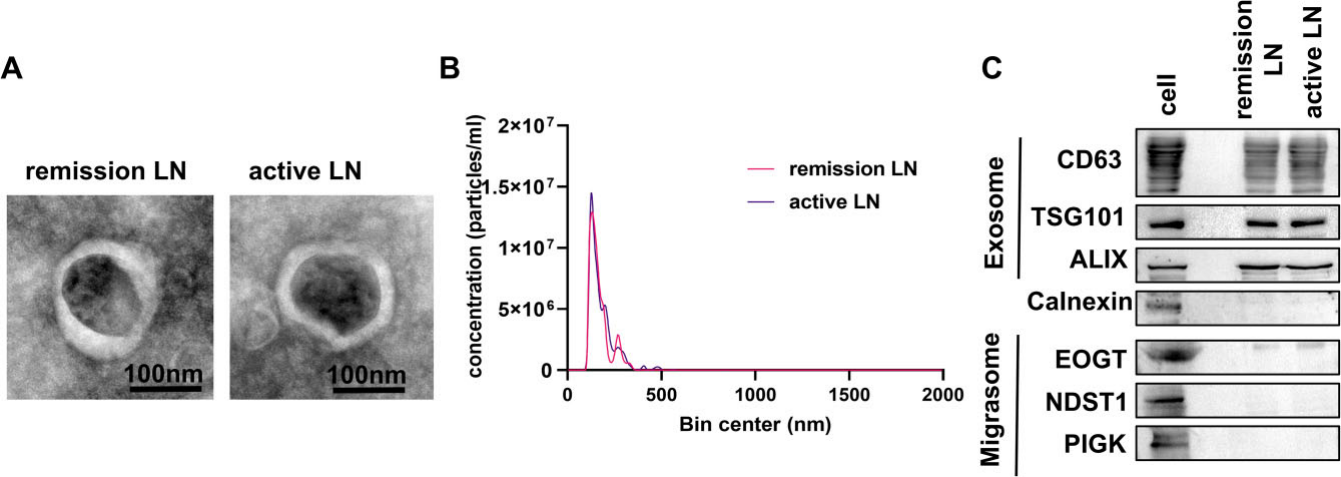
Isolation and characterization of uEVs. (**a**) TEM analysis of uEVs. The scale bar represents 100 nm. (**b**) NTA quantification of urinary EVs. (**c**) Western blotting analysis of urinary EVs.

EVs are enriched in surface glycoconjugates, facilitating selective binding to lectins such as WGA [7–10]. We and Li *et al.* [10] previously established an efficient and user-friendly method for EV isolation using WGA-conjugated magnetic beads, which has demonstrated utility in biomarker studies across chronic kidney disease, breast cancer, and fatty liver disease [7–10]. In the present study, this approach was applied to isolate urinary EVs from patients with SLE, including those with active and remission LN. Isolated EVs were characterized using TEM, NTA, and western blotting. As previously reported [7–10], the EVs displayed the typical bilayer membrane morphology and a mean particle size of ∼100 nm (Fig. 1a and b). Western blot analysis confirmed the presence of established EV markers TSG101, CD63, and ALIX-while CALNEXIN, an endoplasmic reticulum protein and negative EV marker that was detected only in whole-cell lysates and not in the EV fraction (Fig. 1c).

### Expression profile of long RNA fragments in uEVs from SLE patients with active or remission LN

**Figure 2:**
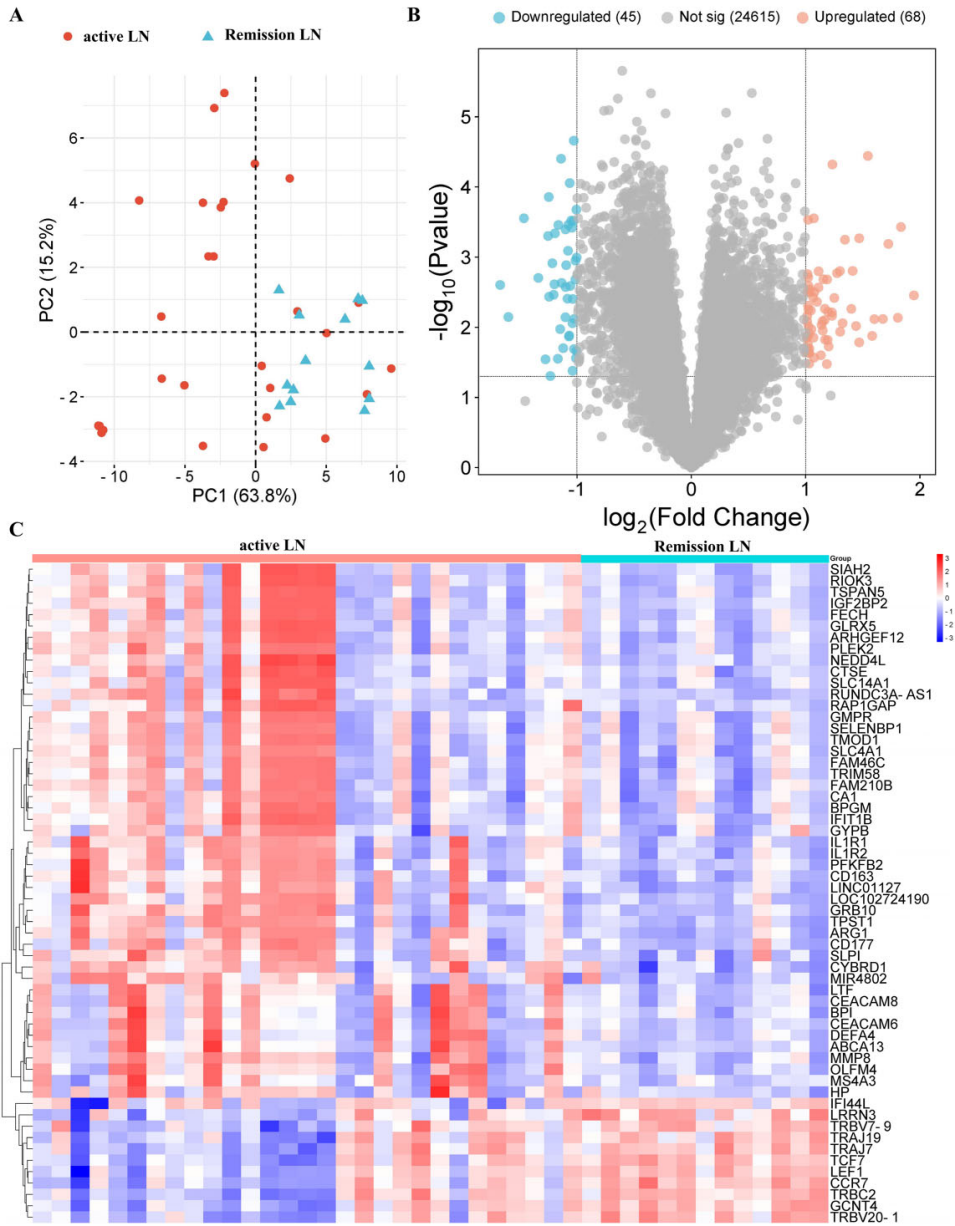
Principal component (PC) analysis (**a**), volcano plot (**b**) and heatmap (**c**) analysis of RNA sequencing data of urinary EVs from SLE patients with active LN and remission LN.

To examine the long RNA expression profiles of uEVs from patients with SLE and active versus remission LN, total RNA was extracted from EVs isolated using WGA-conjugated magnetic beads and subjected to RNA sequencing. Principal component analysis revealed a clear separation between the active and remission LN groups (Fig. 2a). Differential expression analysis identified 113 dysregulated long RNAs, including 68 upregulated and 45 downregulated transcripts (Fig. 2b). Hierarchical clustering and heatmap visualization further confirmed distinct transcriptomic signatures associated with each clinical state (Fig. 2c).

### Gene ontology and KEGG enrichment analyses of the dysregulated RNAs in uEVs

**Figure 3:**
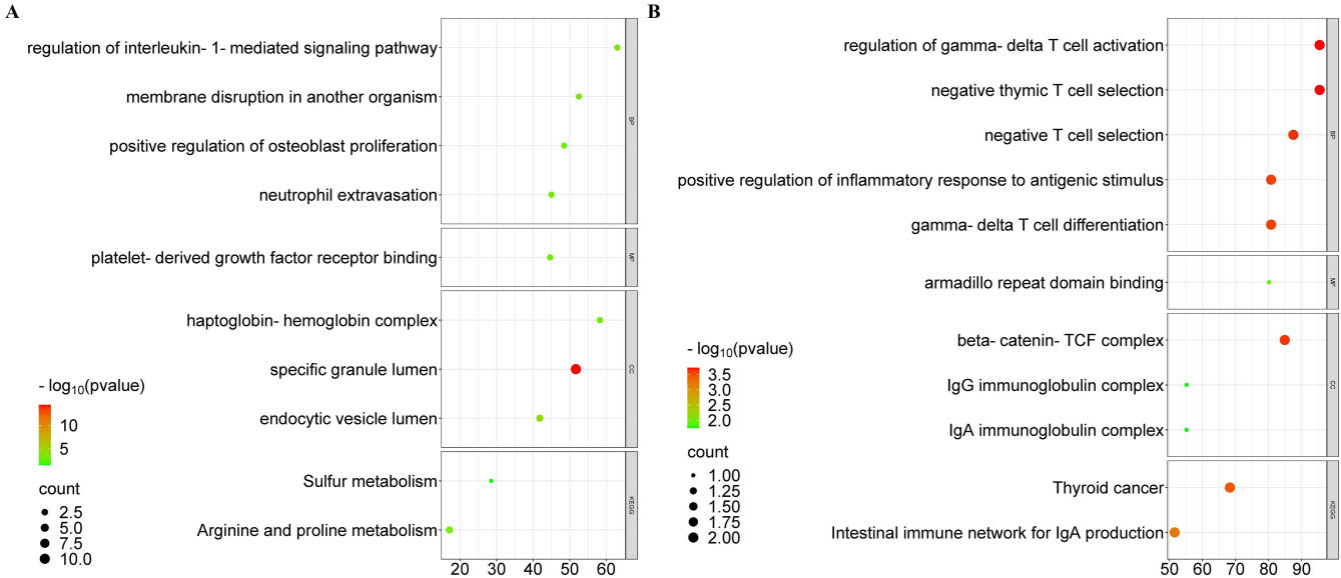
GO and KEGG analyses of upregulated (**a**) and downregulated (**b**) RNAs.

Gene ontology (GO) and Kyoto Encyclopedia of Genes and Genomes (KEGG) pathway analyses were conducted to elucidate the functional relevance of dysregulated RNAs in uEVs. Pathway enrichment using the PANTHER database revealed that upregulated RNAs were predominantly associated with immune-related processes characteristic of active LN, including interleukin-1-mediated signaling and neutrophil extravasation (Fig. 3a). By contrast, downregulated RNAs were enriched in pathways linked to T-cell regulation, such as γδ T-cell activation and thymic negative selection (Fig. 3b). These findings suggest that uEV-derived RNAs may contribute to the dysregulation of inflammatory pathways implicated in LN pathogenesis.

### Biomarker validation of RNAs in urinary EVs for active LN

**Figure 4:**
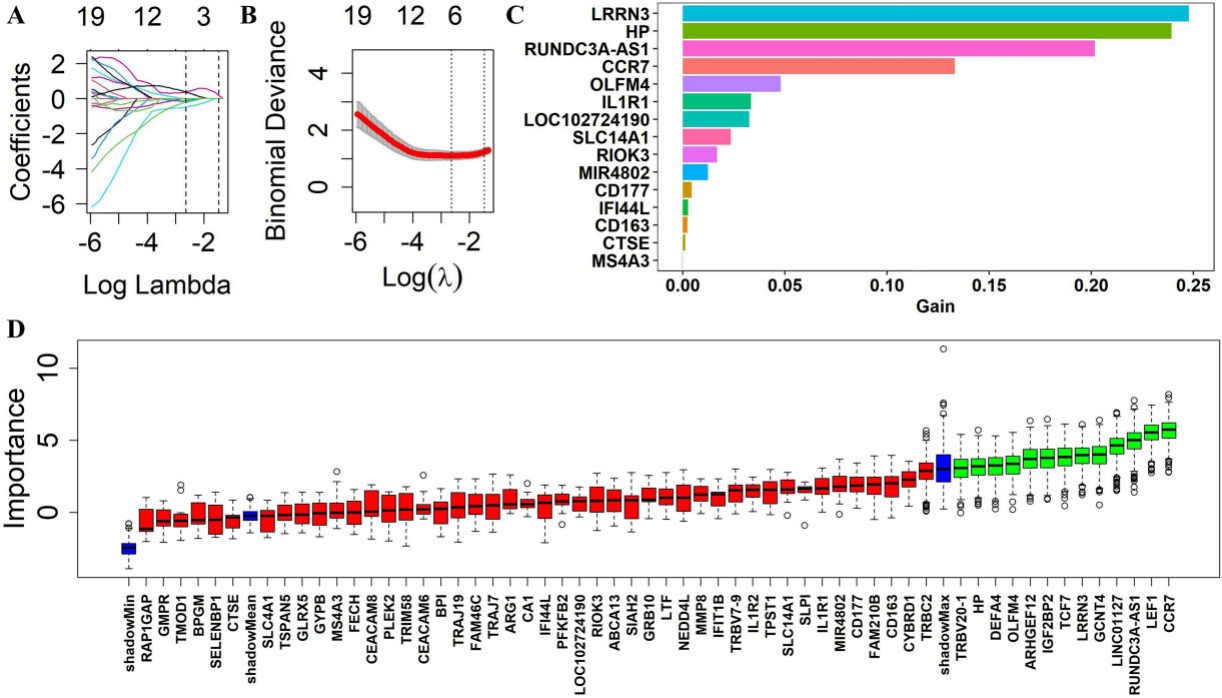
LASSO (**a** and **b**), XGBoost (**c**), and Boruta (**d**) were used to assess the diagnostic potential of the 113 dysregulated RNAs identified in urinary EVs for the detection of active LN.

To assess the diagnostic utility of the 113 dysregulated RNAs identified in uEVs for active LN, three machine learning algorithms—LASSO [11], XGBoost [12], and Boruta [13]—were applied. LASSO identified six candidate biomarkers (RIOK3, CCR7, LINC01127, RUNDC3A-AS1, ABCA13, and LRRN3; Fig. 4a and b), while XGBoost and Boruta identified 15 and 13 candidates, respectively (Fig. 4c and d). Cross-method comparison revealed four consistently selected biomarkers: two mRNAs (CCR7 and LRRN3) and two long noncoding RNAs (LINC01127 and RUNDC3A-AS1).

**Figure 5:**
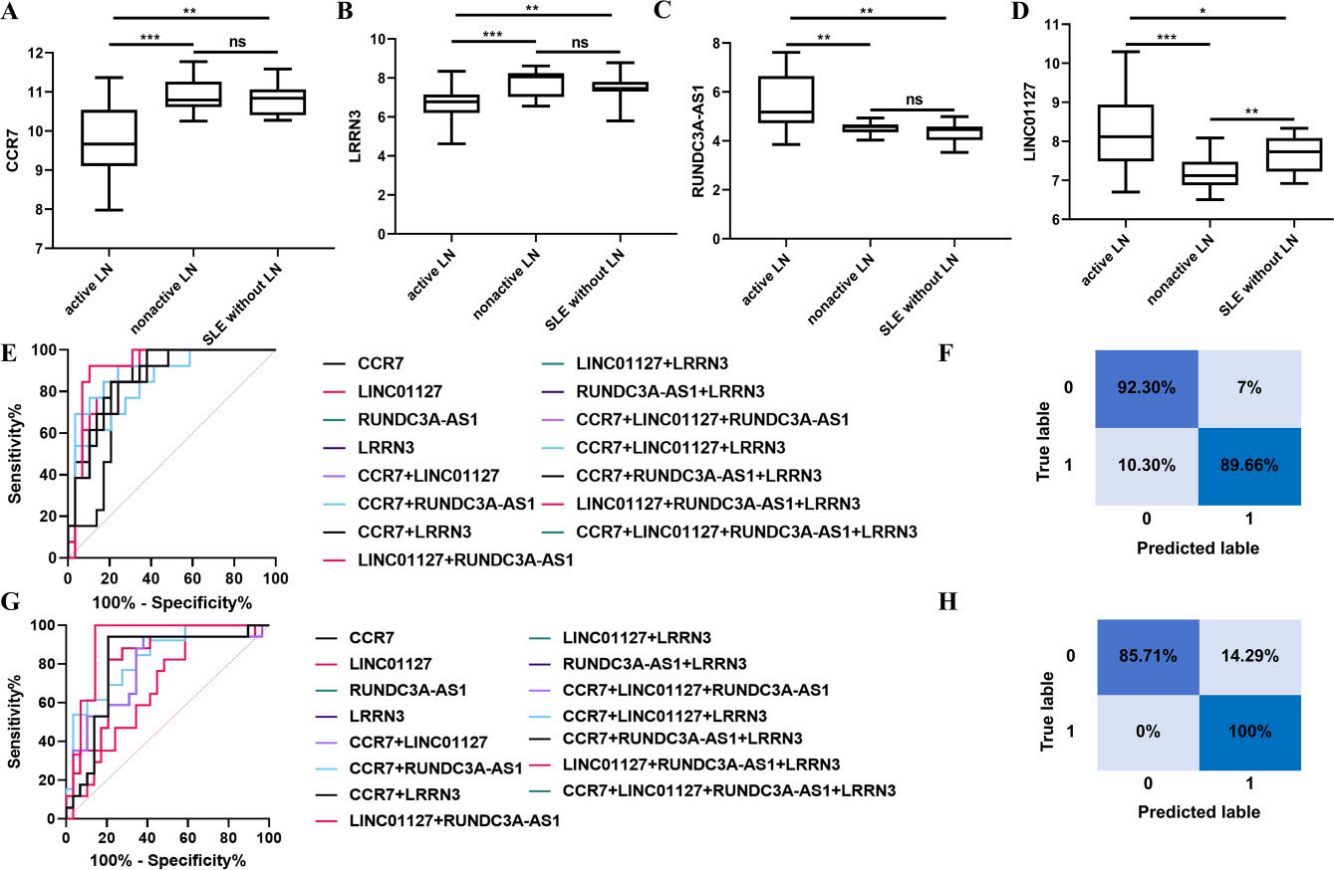
RNAs contained in urinary EVs used as markers for diagnosing LN. (**a–d**) CCR7 (a), LRRN3 (b), RUNDC3A-AS1 (c), and LINC01127 (d) in urinary EVs from SLE patients with active LN, remission LN, and without LN. (**e**) ROC curve analysis of the combination of CCR7, LRRN3, RUNDC3A-AS1, and LINC01127 in urinary EVs to distinguish SLE patients with active LNs from those with remission LNs. (**f**) Confusion matrix of LRRN3, RUNDC3A-AS1, and LINC01127 combined in uEVs to distinguish SLE patients with active LNs from those with remission LNs. (**g**) ROC curve analysis of the combination of CCR7, LRRN3, RUNDC3A-AS1, and LINC01127 in urinary EVs to distinguish SLE patients with active LNs from those with non-LNs. (**h**) Confusion matrix of LRRN3, RUNDC3A-AS1, and LINC01127 combined in uEVs to distinguish SLE patients with active LNs from those with non-LNs. The data from three independent experiments (*n* = 3) are presented as the means ± SDs. Not significant, *P* > .05; *P < .05;**P < .01; ***P < .001.

The four candidate biomarkers were validated in an independent cohort of 143 patients with SLE, including 89 with active LN, 41 in remission, and 13 without renal involvement, using RT–qPCR (Table S1). NTA confirmed no significant differences in uEV size or concentration among the groups (Fig. S1). The specificity and amplification efficiency of the primers for CCR7, LINC01127, RUNDC3A-AS1, and LRRN3 were validated by RT–qPCR using synthetic RNA templates (Fig. S2). Expression analysis showed that CCR7 and LRRN3 were significantly downregulated in active LN compared with both remission LN and non-LN SLE patients (Fig. 5a and b). RUNDC3A-AS1 was markedly upregulated in active LN, with no significant differences between remission and non-LN groups (Fig. 5c). LINC01127 was also elevated in active LN but was reduced in remission LN relative to non-LN SLE patients (Fig. 5d). Moreover, the expression levels of CCR7, LINC01127, RUNDC3A-AS1, and LRRN3 were also measured in uEVs from healthy controls. No significant differences in CCR7, LINC01127, or LRRN3 expression were observed between healthy controls, patients in LN remission, and non-LN SLE patients. By contrast, RUNDC3A-AS1 expression was significantly elevated in both active LN and non-LN SLE patients compared to healthy controls (Fig. S3).

Receiver operating characteristic (ROC) curve analysis demonstrated that all four biomarkers discriminated active LN from remission, each with an area under the curve (AUC) >0.80 (Table S2). A logistic regression model combining LINC01127, RUNDC3A-AS1, and LRRN3 yielded the highest diagnostic performance, with an AUC of 0.926, sensitivity of 89.66%, and specificity of 92.30% (Fig. 5e and f; Table S2). This model also performed well in distinguishing active LN from non-LN SLE patients, achieving an AUC of 0.915, sensitivity of 100%, and specificity of 85.71% (Fig. 5g and h; Table S3). Accordingly, LINC01127, RUNDC3A-AS1, and LRRN3 were selected for further investigation.

### Correlation analysis between LINC01127, RUNDC3A-AS1, and LRRN3 expression patterns in uEVs and histological features

**Figure 6:**
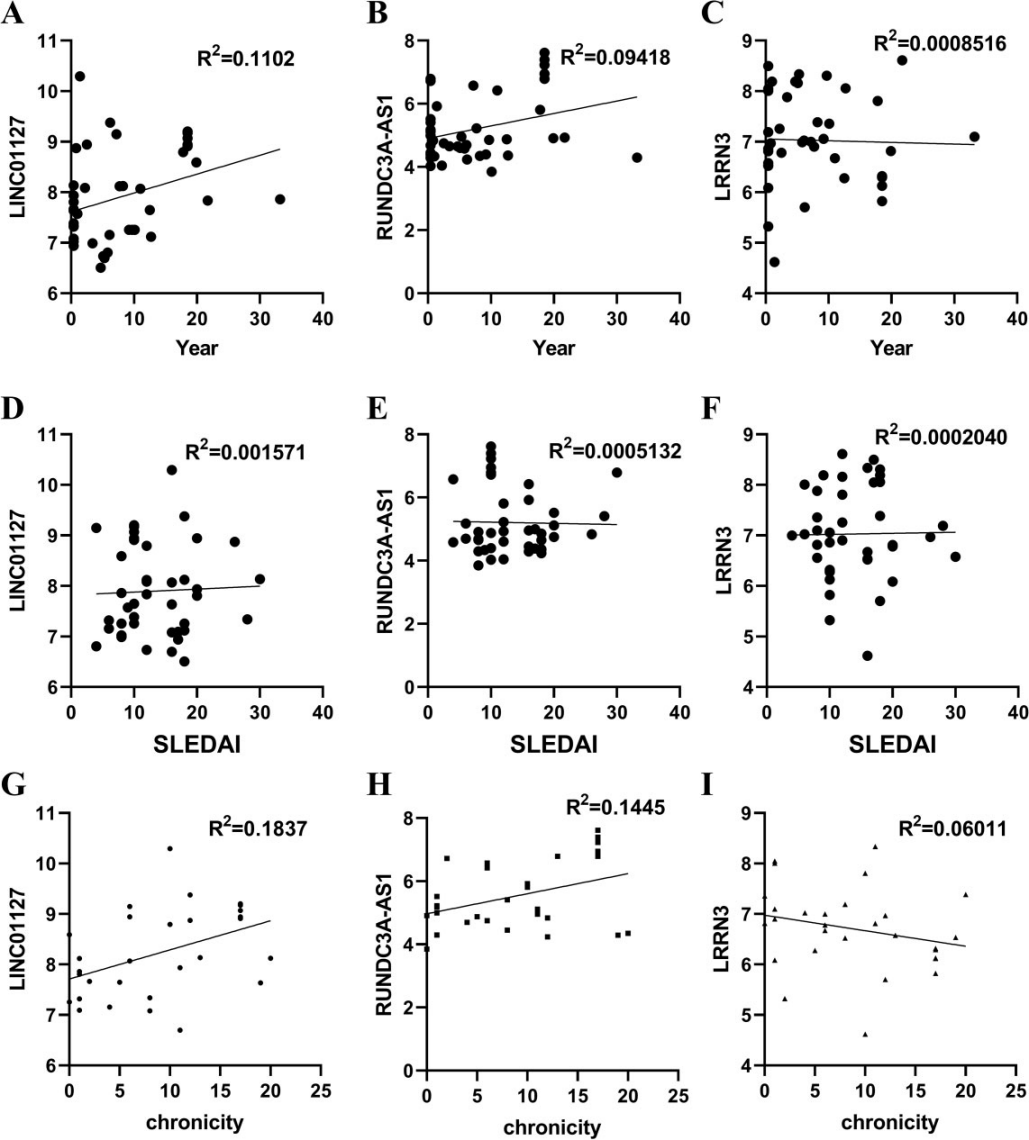
Correlations between LRRN3, RUNDC3A-AS1, and LINC01127 and duration (**a–c**), SLEDAI (**d–f**), and chronicity (**g–i**). The data from three independent experiments (*n* = 3) are presented as the means ± SDs.

To investigate the association between uEV-derived LINC01127, RUNDC3A-AS1, and LRRN3 expression and LN pathology, correlation analyses were performed. As shown in Fig. 6a–f, none of the three biomarkers correlated with disease duration or the SLE Disease Activity Index (SLEDAI). However, LINC01127 and RUNDC3A-AS1 levels were positively associated with the CI, while LRRN3 expression was negatively correlated (Fig. 6g–i).

**Figure 7:**
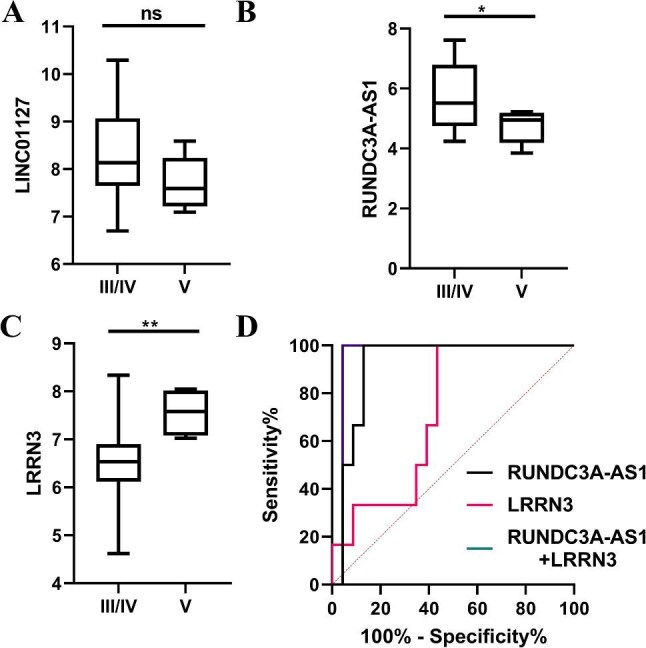
RNAs contained in urinary EVs used as markers for distinguishing between proliferative (class III/IV) and isolated membranous LN-A (class V) cells. (**a–c**) LINC01127 (a), RUNDC3A-AS1 (**b**), and LRRN3 (**c**) in urinary EVs from class III/IV- and V-activated LNs. (**d**) ROC curve analysis of LRRN3 and RUNDC3A-AS1 in urinary EVs to distinguish class III/IV activated LNs from class V-activated LNs. The data from three independent experiments (*n* = 3) are presented as the means ± SDs. Not significant, *P* > .05; **P < .01.

Given the clinical importance of differentiating proliferative LN (class III/IV) from isolated membranous LN (class V) to guide therapeutic decisions, we next assessed the discriminatory capacity of these biomarkers. LINC01127 expression did not differ significantly between proliferative and membranous LN (Fig. 7a). By contrast, RUNDC3A-AS1 was significantly upregulated and LRRN3 significantly downregulated in class III/IV compared to class V lesions (Fig. 7b and c). ROC curve analysis confirmed their diagnostic value, with AUCs of 0.9203 for RUNDC3A-AS1 and 0.7174 for LRRN3 (Fig. 7d; Table S4). A combined logistic regression model incorporating both markers achieved the highest diagnostic accuracy, with an AUC of 0.9565, 100% sensitivity, and 95.65% specificity (Fig. 7d; Table S4).

## DISCUSSION

SLE is a chronic autoimmune disease with multisystem involvement, among which LN represents one of the most severe manifestations, affecting ∼20%–70% of patients. Despite therapeutic advances, up to 10% of individuals with LN progress to ESKD within 10 years [16]. Clinical management relies heavily on histopathological classification, primarily to distinguish active from remission states [15]. Patients in remission are typically managed with antimalarials and renin–angiotensin–aldosterone system inhibitors, while those with active LN require intensive immunosuppressive induction therapy followed by maintenance regimens to prevent relapse [15, 17]. However, conventional clinical indicators—such as proteinuria, serum creatinine, and serological markers—correlate poorly with underlying renal pathology [18]. Currently, no validated noninvasive biomarkers are available to assess histological activity or guide therapeutic decisions [18]. As a result, kidney biopsy remains the gold standard for evaluating suspected LN flares, despite its inherent risks, including pain, macroscopic hematuria, hematoma, nephrectomy, and, in rare cases, mortality [18–21]. These limitations highlight the urgent need for reliable, noninvasive biomarkers capable of accurately reflecting renal pathology, predicting treatment response, and informing long-term prognosis in LN.

Urinary biomarkers offer valuable insights into glomerular and tubular pathology, reflecting processes such as inflammation, fibrosis, and active lesions [22]. Owing to its noninvasive nature and ease of collection, urine is an ideal source for biomarker discovery. Among urinary components, EVs have emerged as promising indicators of renal dysfunction. Our previous work identified elevated levels of tRNA-derived small noncoding RNAs—specifically tRF3-Ile-AAT-1 and tiRNA5-Lys-CTT-1—in urinary exosomes from patients with LN, supporting their diagnostic potential [23]. Other studies have reported inverse correlations between urinary exosomal miR-29c and histological chronicity, as well as between miR-3201/miR-1273e and intracapillary inflammation [24, 25]. Additional miRNAs, including miR-146a, miR-26a, and miR-30b, have been associated with disease activity and renal injury through modulation of inflammatory and fibrotic pathways [12, 26]. While small RNAs have been extensively studied, long RNA species within EVs remain underexplored, despite several advantages. Long RNAs typically demonstrate greater tissue specificity and are more readily detected using standard RT–qPCR, unlike small RNAs, which require complex and costly amplification protocols. These characteristics position long RNAs as attractive candidates for clinical diagnostics. However, few studies have assessed their diagnostic value in blood or urine from LN patients [27, 28]. In the present study, we established a robust platform integrating WGA, magnetic bead-based EV isolation, and RT–qPCR to quantify long RNAs in uEVs from SLE patients. We identified three long RNAs—LINC01127, RUNDC3A-AS1, and LRRN3—as potential biomarkers of active LN. Notably, RUNDC3A-AS1 and LRRN3 also differentiated between proliferative (class III/IV) and membranous (class V) LN.

LINC01127 has been shown to activate the ERK and AKT signaling pathways and regulate MAP4K4 to stimulate the JNK pathway-mechanisms implicated in both the pathogenesis of LN and response to therapy [29, 30]. RUNDC3A-AS1 has been linked to oncogenic pathways, including the miR-182–5p/ADAM9 and miR-151b/SNRPB axes, and is associated with processes such as cuproptosis and tissue fibrosis [31–35]. Of note, ADAM9 plays a role in TGF-β signaling and Th17 cell differentiation, both of which are relevant to LN pathophysiology [34, 35]. LRRN3, a member of the NOD-like receptor family, is known to promote proinflammatory cytokine expression and may contribute to renal inflammation [36, 37]. These functional associations support the potential mechanistic relevance of these long RNAs in LN.

Alterations in glycosylation patterns and glycocalyx integrity are well-documented features of kidney diseases, including LN, and may influence the glycoprotein composition of EVs, potentially introducing bias in glycan-dependent isolation methods such as WGA-based capture [38]. In this study, we employed WGA-conjugated magnetic beads as a practical and efficient approach to isolate a broad population of uEVs. WGA specifically binds to N-acetylglucosamine and sialic acid residues, which are widely expressed on the surface of many EVs. While this strategy may preferentially enrich for vesicles with enhanced surface glycosylation—a feature that may vary with disease state—it is important to emphasize two points: (i) our primary goal was biomarker discovery, not comprehensive EV profiling. The focus of this work was to identify noninvasive, disease-associated RNA signatures in uEVs, rather than to characterize the full diversity of EV subpopulations. Even if WGA-based enrichment reflects disease-modified glycosylation, such alterations are part of the pathological process and may actually increase the clinical relevance of captured biomarkers. (ii) Enrichment for disease-altered EVs may enhance diagnostic performance. Pathological processes such as glycocalyx shedding and renal fibrosis are known to modify EV surface glycosylation. WGA-based isolation may therefore act as a disease-contextual ‘filter’, selectively capturing vesicles that reflect active renal injury. This may enhance sensitivity to disease-specific molecular signals, making it a useful feature rather than a methodological flaw in the context of diagnostic biomarker development.

Despite these promising findings, the present study is limited by its relatively small sample size. Validation in larger, multicenter cohorts will be essential to confirm their diagnostic and prognostic utility. Moreover, the functional roles of these RNAs within urinary EVs remain to be elucidated. Notably, some patients with clinically quiescent disease exhibited RNA expression profiles similar to those with active LN (Fig. 2), raising the possibility of subclinical activity or imminent flare. Longitudinal follow-up of these individuals is ongoing to better understand the temporal dynamics of these biomarkers.

In conclusion, we identified three long RNAs in urinary EVs-LINC01127, RUNDC3A-AS1, and LRRN3-as potential noninvasive biomarkers for active LN. Importantly, RUNDC3A-AS1 and LRRN3 also demonstrated utility in distinguishing proliferative from membranous LN, underscoring their potential role in guiding clinical decision-making and personalized treatment strategies.

## Supplementary Material

sfaf295_Supplemental_File

## Data Availability

All the data are available in the main text or supplementary material. Detailed raw data and information are provided by the corresponding author upon request.
