# Supplementary material for: Urinary extracellular vesicle RNAs as novel biomarkers for diagnosis and prognosis of lupus nephritis
Source: Clin Kidney J. 2025 Sep 22;18(10):sfaf295. doi: 10.1093/ckj/sfaf295 (PMC12527279; doi:10.1093/ckj/sfaf295)

**Table S1. Demographics and clinical characteristics of the study participants.**

|  |  | **active LN** | **remission LN** | **without LN** | **HC** |
| --- | --- | --- | --- | --- | --- |
| **Numer** |  | 89 | 41 | 13 | 20 |
| **age** |  | 35.27(10.58) | 29.46(9.01) | 32.65(6.54) | 30(5.91) |
| **female(%)** |  | 75.86 | 92.31 | 82.35 | 75.00 |
| **disease duration** |  | 8.48(8.68) | 5.90(6.18) |  |  |
| **sledai** |  | 14.17(8.68) | 12.00(4.53) |  |  |
| **biopsy class(%)** |  |  |  |  |  |
|  | **III/IV** | 0.79 |  |  |  |
|  | **V** | 0.21 |  |  |  |

**Table S2. ROC curve analysis of LINC01127, RUNDC3A-AS1 and LRRN3 in active LN and remission LN.**

| **Gene** | **AUC** |
| --- | --- |
| **CCR7** | 0.873 |
| **LINC01127** | 0.851 |
| **RUNDC3A-AS1** | 0.812 |
| **LRRN3** | 0.838 |
| **CCR7+LINC01127** | 0.873 |
| **CCR7+RUNDC3A-AS1** | 0.873 |
| **CCR7+LRRN3** | 0.873 |
| **LINC01127+RUNDC3A-AS1** | 0.897 |
| **LINC01127+LRRN3** | 0.851 |
| **RUNDC3A-AS1+LRRN3** | 0.912 |
| **CCR7+LINC01127+RUNDC3A-AS1** | 0.873 |
| **CCR7+LINC01127+LRRN3** | 0.873 |
| **CCR7+RUNDC3A-AS1+LRRN3** | 0.873 |
| **LINC01127+RUNDC3A-AS1+LRRN3** | 0.926 |
| **CCR7+LINC01127+RUNDC3A-AS1+LRRN3** | 0.873 |

**Table S3. ROC curve analysis of LINC01127, RUNDC3A-AS1 and LRRN3 in active LN and non-LN.**

| **Gene** | **AUC** |
| --- | --- |
| **CCR7** | 0.781 |
| **LINC01127** | 0.686 |
| **RUNDC3A-AS1** | 0.799 |
| **LRRN3** | 0.809 |
| **CCR7+LINC01127** | 0.781 |
| **CCR7+RUNDC3A-AS1** | 0.781 |
| **CCR7+LRRN3** | 0.809 |
| **LINC01127+RUNDC3A-AS1** | 0.799 |
| **LINC01127+LRRN3** | 0.809 |
| **RUNDC3A-AS1+LRRN3** | 0.809 |
| **CCR7+LINC01127+RUNDC3A-AS1** | 0.781 |
| **CCR7+LINC01127+LRRN3** | 0.809 |
| **CCR7+RUNDC3A-AS1+LRRN3** | 0.809 |
| **LINC01127+RUNDC3A-AS1+LRRN3** | 0.915 |
| **CCR7+LINC01127+RUNDC3A-AS1+LRRN3** | 0.809 |

**Table S4. ROC curve analysis of RUNDC3A-AS1 and LRRN3 for classes III/IV and class V. ***: p<0.001**

| **Gene** | **AUC** |
| --- | --- |
| **RUNDC3A-AS1** | 0.9203*** |
| **LRRN3** | 0.7174*** |
| **RUNDC3A-AS1+LRRN3** | 0.9565*** |

**Figure S1. The concentration and size of EVs in the urine of SLE patients with active LN, remission LN and without LN.**


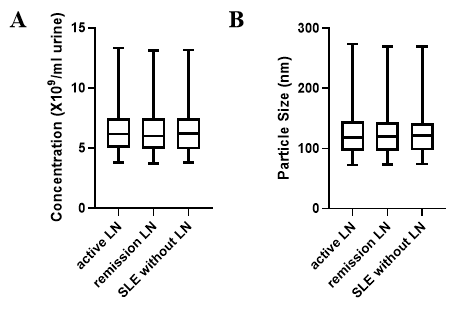


**Figure S2. (A-D) Standard curves for absolute quantification by RT-qPCR of synthetic CCR7 (A), LRRN3 (B), RUNDC3A-AS1 (C) and LINC01127 (D).
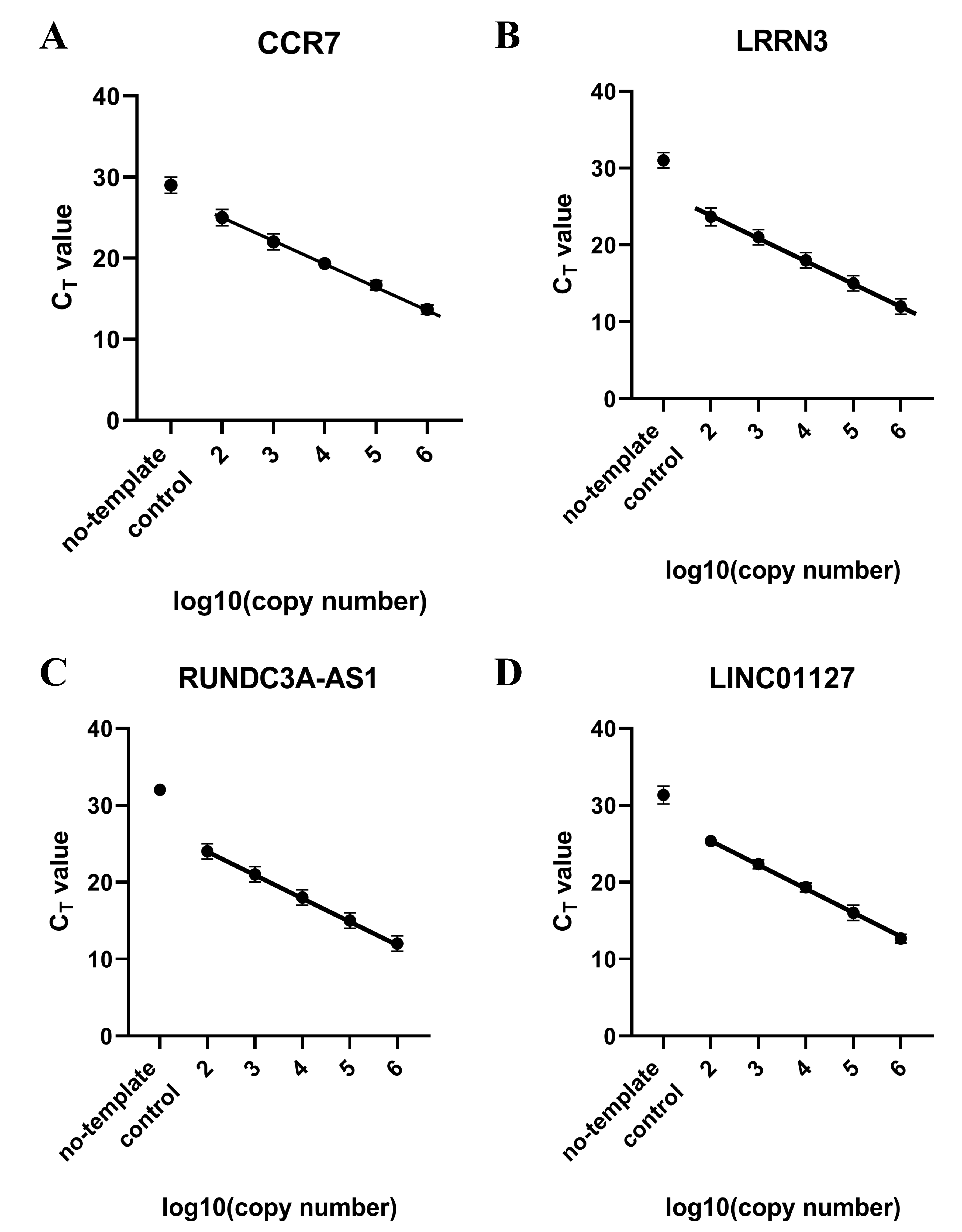
**

**Figure S3. (A-D) CCR7 (A), LRRN3 (B), RUNDC3A-AS1 (C) and LINC01127 (D) in urinary EVs from healthy controls (HC) and SLE patients with active LN, remission LN and without LN.**


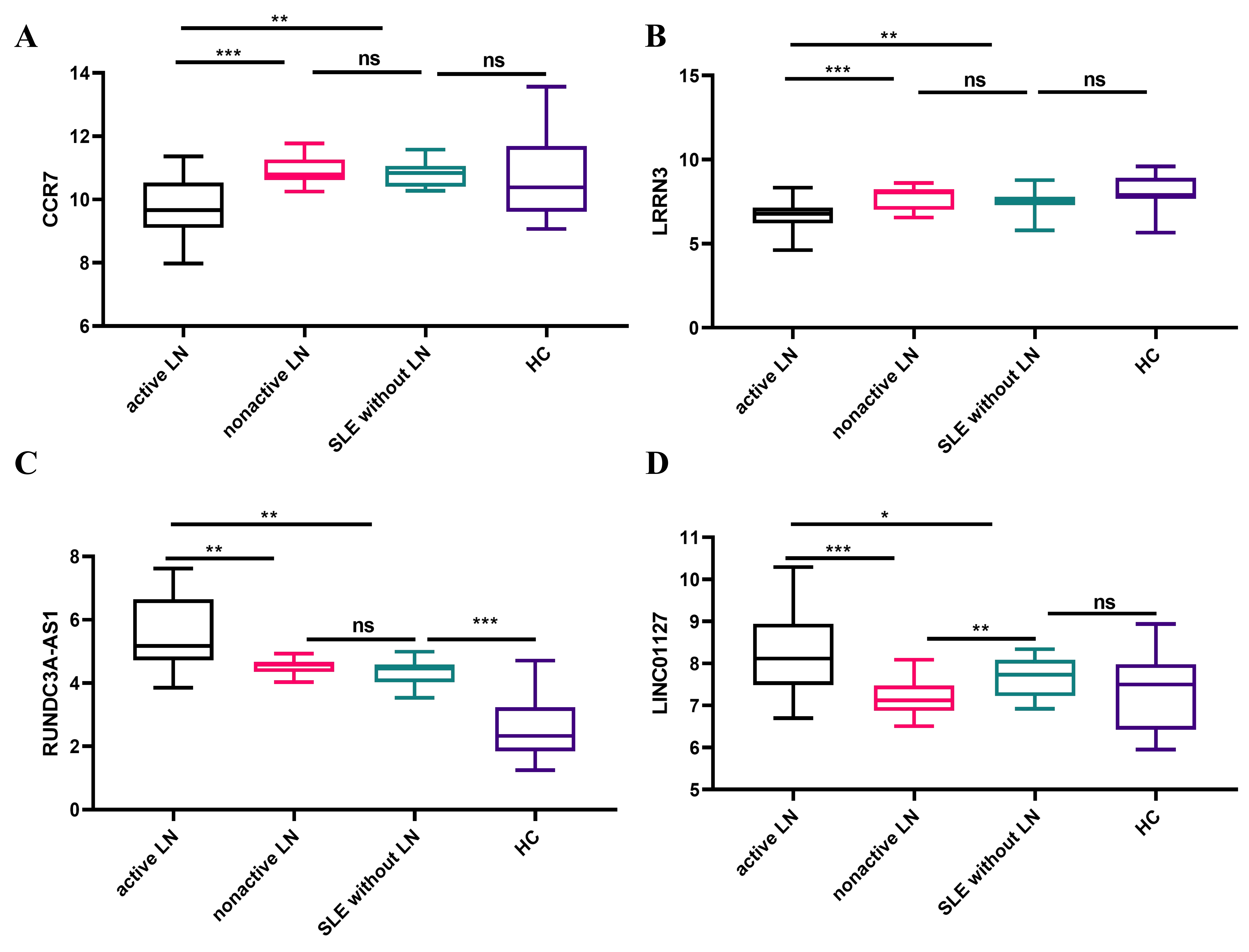

Supplement: sfaf295_Supplemental_File [file sfaf295_supplemental_file.docx]
